# Изучение точности измерения системы мониторинга уровня глюкозы в крови Джимейт ЛАЙФ

**DOI:** 10.14341/probl13627

**Published:** 2026-01-18

**Authors:** В. А. Быбин, Н. М. Быкова, Е. А. Харитонова

**Affiliations:** Общество с ограниченной ответственностью «ДИАТЕК»Россия; Limited Liability Company «DIATEK»Russian Federation; Иркутская городская клиническая больница №10Россия; Irkutsk City Clinical Hospital No. 10Russian Federation

**Keywords:** сахарный диабет, система мониторинга уровня глюкозы в крови, глюкометр, тест-полоска, точность системы, diabetes mellitus, blood glucose self-monitoring, reproducibility of results

## Abstract

**ОБОСНОВАНИЕ:**

ОБОСНОВАНИЕ. В настоящее время сахарный диабет изучают как актуальную социальную проблему. Многие диабетогенные факторы, то есть воздействия, способные запустить развитие у человека сахарного диабета, имеют социальную природу или проистекают из социальных обстоятельств. Среди причин, способствующих развитию этого патологического состояния, можно выделить: неправильное питание, стресс, малоподвижный образ жизни, ухудшение экологической обстановки, вирусные инфекции и т.д. На данный момент существует множество систем мониторинга уровня глюкозы в крови, отличающихся по точности измерений, которая важна не только для ежедневного наблюдения, экстренного измерения при ухудшении самочувствия, но и для подбора адекватной дозы инсулина. С появлением нового продукта на потребительском рынке, в частности системы мониторинга глюкозы в крови Джимейт ЛАЙФ, к нему возникает много вопросов: настолько ли надежен и точен прибор, как об этом заявляет производитель?

**ЦЕЛЬ:**

ЦЕЛЬ. Изучить соответствие точности измерения отечественной системы мониторинга глюкозы в крови Джимейт ЛАЙФ требованиям ГОСТ Р ИСО 15197-2015 «Тест-системы для диагностики in vitro. Требования к системам мониторинга глюкозы в крови для самоконтроля при лечении сахарного диабета» (идентичен Международному ISO 15197:2015).

**МАТЕРИАЛЫ И МЕТОДЫ:**

МАТЕРИАЛЫ И МЕТОДЫ. Для дизайна экспериментов по изучению точности измерения системы мониторинга уровня глюкозы Джимейт ЛАЙФ руководствовались требованиям ГОСТ Р ИСО 15197-2015. Точность системы изучали на образцах капиллярной крови. Образцы капиллярной крови брали у здоровых людей, а также у амбулаторных пациентов с гипогликемическими и гипергликемическими состояниями в эндокринологическом отделении ОГАУЗ «ИГКБ № 10» г. Иркутска. Исследования проводились в период с 05.03.2025 по 06.03.2025 гг. в эндокринологическом отделении ОГАУЗ «ИГКБ № 10» г. Иркутска.

**РЕЗУЛЬТАТЫ:**

РЕЗУЛЬТАТЫ. Для проведения оценки точности системы протестировали 600 образцов капиллярной крови, полученных от добровольцев, — в основном амбулаторных пациентов с сахарным диабетом или больных в стационаре. Результаты соответствовали минимальным критериям точности стандарта ГОСТ Р ИСО 15197-2015.

**ЗАКЛЮЧЕНИЕ:**

ЗАКЛЮЧЕНИЕ. Результат тестов на измерение уровня глюкозы в крови зависит от технологичности и качества глюкометра и тест-полосок. Система должна соответствовать минимальным требованиям стандарта ГОСТ Р ИСО 15197-2015. Тестирование системы Джимейт ЛАЙФ показало, что результаты по проверке точности системы мониторинга глюкозы Джимейт ЛАЙФ лучше, чем указано в требованиях.

## Abstract (en)

**BACKGROUND:**

BACKGROUND. Currently, diabetes mellitus is studied as an urgent social problem. This is due to the fact that the number of identified cases is constantly growing, the course of the disease often becomes chronic, various complications develop, which lead to a deterioration in the quality of life and a reduction in its duration. At the moment, there are many blood glucose monitoring systems that differ in measurement accuracy, which is important not only for daily monitoring, emergency measurement when health deteriorates, but also for selecting an adequate dose of insulin. With the advent of a new product on the consumer market, in particular the Gmate LIFE blood glucose monitoring system, many questions arise — is the device as reliable and accurate as the manufacturer claims?

**AIM:**

AIM. To evaluate whether the measurement accuracy of the domestic Gmate LIFE blood glucose monitoring system meets the requirements of GOST R ISO 15197-2015 «In vitro diagnostic test systems. Requirements for blood glucose monitoring systems for self-testing in managing diabetes mellitus» (identical to International ISO 15197:2013).

**MATERIALS AND METHODS:**

MATERIALS AND METHODS. The design of experiments to study the measurement accuracy of the Gmate LIFE glucose monitoring system was guided by the requirements of GOST R ISO 15197-2015. The accuracy of the system was studied on capillary blood samples. Capillary blood samples were taken from healthy individuals, as well as from outpatients with hypoglycemic and hyperglycemic conditions in the endocrinology department of the Irkutsk Regional State Autonomous Healthcare Institution «Irkutsk City Clinical Hospital No. 10». The studies were conducted from 05.03.2025 to 06.03.2025 in the endocrinology department of the Irkutsk Regional State Autonomous Healthcare Institution «Irkutsk City Clinical Hospital No. 10».

**RESULTS:**

RESULTS. To evaluate the accuracy of the system, 600 capillary blood samples were tested, obtained from volunteers — mainly outpatients with diabetes mellitus or inpatients. The results met the minimum accuracy criteria of the GOST R ISO 15197-2015 standard.

**CONCLUSION:**

CONCLUSION. The result of tests for measuring blood glucose levels depends on the technology and quality of the glucometer and test strips. The system must meet the minimum requirements of the GOST R ISO 15197-2015 standard. Testing of the Gmate LIFE system showed that the results of accuracy of the Gmate LIFE glucose monitoring system fully comply with the standard, and according to the system accuracy criterion, they show a result better than specified in the requirements.

## ОБОСНОВАНИЕ

Сахарный диабет (СД) входит в число самых распространенных в мире хронических заболеваний и занимает третье место после сердечно-сосудистых и онкологических заболеваний среди непосредственных причин смерти [1].

В настоящее время СД изучают как актуальную социальную проблему. Многие диабетогенные факторы, то есть воздействия, способные запустить развитие у человека СД, имеют социальную природу или проистекают из социальных обстоятельств. Социальные проблемы не только способствуют возникновению СД, но и сопровождают страдающих этим недугом людей на протяжении жизни. В свою очередь СД порождает новые медицинские и социальные проблемы [2].

Среди причин, способствующих развитию этого патологического состояния, можно выделить: неправильное питание, стресс, малоподвижный образ жизни, ухудшение экологической обстановки, вирусные инфекции и т.д. Перечисленные факторы влияют в основном на развитие СД 2 типа (СД2), при котором происходит нарушение углеводного обмена, вызванное преимущественной инсулинорезистентностью и относительной инсулиновой недостаточностью или преимущественным нарушением секреции инсулина с инсулинорезистентностью или без нее. Лечение в этом случае предусматривает соблюдение диеты, выполнение умеренных физических нагрузок, терапию лекарственными препаратами и постоянный самоконтроль гликемии. СД 1 типа (СД1) может развиваться с раннего возраста, в основе его лежит деструкция β-клеток поджелудочной железы, в результате развивается выраженный дефицит инсулина [3]. Помимо распространенных двух типов сахарного диабета существуют специфические формы, которые также клинически проявляются гипергликемией и мочеизнурением: диабет, индуцированный лекарствами или инфекциями; генетические синдромы, сочетающиеся с сахарным диабетом и др.), и гестационный СД, возникающий на фоне беременности у некоторых женщин [4].

Несмотря на то, что в последнее время наблюдается тенденция к поддержанию здорового образа жизни, число заболевших и количество людей, попадающих в группу риска, растет. Рост идет преимущественно в странах со средним и низким уровнем доходов населения. В Российской Федерации (РФ), как и в других странах мира, отмечается прирост распространенности СД эпидемического характера: с 2000 г. численность пациентов с СД увеличилась более чем в 2 раза. По данным Федеральной службы государственной статистики (Росстат) и Федерального государственного бюджетного учреждения «Центральный научно-исследовательский институт организации и информатизации здравоохранения» Министерства здравоохранения РФ (ЦНИИОИЗ), прирост количества пациентов с СД за период 2009–2023 гг. составил 74,5%; общее количество пациентов с СД в РФ на 01.01.2024 г. насчитывает 5 547 879 человек, из них подавляющее большинство взрослые с СД2 — 5 168 374 и 349 338 — пациенты с СД1, из них взрослые — 288 020 человек и дети (до 18 лет) — 61 318 человек [5]. Поэтому очень важно на ранних этапах выявить заболевание, чтобы предотвратить его развитие и появление осложнений. Одним из важнейших средств для профилактики и лечения СД является система мониторинга уровня глюкозы в крови, которая состоит из глюкометра и тест-полосок. Глюкометр — это прибор, регистрирующий и обрабатывающий сигнал, поступающий от тест-полоски, которая, в свою очередь, является сенсором — индикатором уровня глюкозы.

По итогам января — августа 2022 г. лидерами рейтинга по стоимостному объему продаж стали тест-полоски для глюкометра (доля 39,9%) от объема реализации всех диагностических приборов и средств в рублях. По данным Ежемесячного розничного аудита фармацевтического рынка России, проводимого DSM Group (АО «Группа ДСМ»), в период с января по август 2022 г. в сегменте глюкометров лидируют зарубежные компании (Швейцария — удельный вес 47,6% в рублях, США — 21,4%), стоимость которых высока для среднестатистического потребителя. Приобретение тест-полосок к ним также достаточно затратно, так как тест-полоски часто производятся по технологии золотого напыления. Также заметную долю в данном сегменте занимает российская компания (14,3%) [6]. По многочисленным отзывам потребителей, российский производитель предлагает цены ниже, но в результате страдает качество — длительное ожидание результата, снижение точности определения уровня глюкозы и т.д., в то время как потребность в тест-полосках у пациентов, страдающих сахарным диабетом, и, соответственно, затраты на их приобретение, достаточно высоки. Минздравом РФ рекомендовано людям с повышенным содержанием глюкозы в крови производить самоконтроль гликемии не менее четырех раз в день [7].

На сегодняшний день существует широкий спектр различных глюкометров. Однако в основе принципа их работы лежат только два главных метода. Это фото- и электрометрия. Ферменты тест-полосок в фотометрических глюкометрах вступают в реакцию с глюкозой исследуемого образца крови, в результате чего цвет тестовой зоны изменяется пропорционально значению гликемии [8]. Технология фотометрических глюкометров уходит в прошлое. Поэтому далее глубже рассмотрим особенности электрохимических приборов.

Работа электрохимических глюкометров основывается на регистрации электрического импульса, возникающего в результате биохимического превращения глюкозы под действием ферментов [9]. За точность измерения уровня глюкозы в крови электрохимическими глюкометрами отвечает ферментный компонент, используемый в качестве биосенсора в диабетических тест-полосках. Существуют различные ферментные препараты, уникальные по своим свойствам и обладающие разной степенью эффективности, а именно глюкозооксидаза (GOx), пирроло-хинолин-хинон-зависимая глюкозодегидрогеназа (GDH-PQQ), никотин-аденин-динуклеотид-зависимая глюкозодегидрогеназа (GDH-NAD) и флавин-аденин-динуклеотид-зависимая глюкозодегидрогеназа (GDH-FAD). На работу ферментов при измерении содержания глюкозы могут воздействовать другие вещества, к которым чувствителен фермент, и оказывать влияние кислород, растворенный в пробе, а также присутствующий во внешней среде [10].

В технологии, основанной на использовании в качестве биосенсора GOx, большую роль играет кислород: в реакции фермента и глюкозы трансфер электрона осуществляется посредством молекулярного кислорода, а реакция образования перекиси водорода лимитируется содержанием этого элемента в реакционной смеси. Таким образом, при анализе капиллярной, венозной и артериальной крови, обладающие разной степенью насыщенности кислородом, измеряемые значения количества глюкозы у одного человека будут различными [11].

Реакции глюкозадегидрогеназ с глюкозой не подвергаются влиянию кислорода, а перенос электронов происходит за счет естественных или искусственных акцепторов. Одним из представителей этой группы ферментов является GDH-PQQ. Обладая в 25 раз более высокой активностью, чем у GOx, нативный GDH-PQQ имеет некоторые ограничения для чувствительных к глюкозе применений. Наиболее существенным ограничением является его широкая субстратная специфичность, которая может привести к потенциально фатальным ошибкам в восприятии глюкозы в связи с чувствительностью фермента и к другим сахарам, веществам для диализа, некоторым иммуноглобулинам. Из-за такой особенности фермента теряется возможность вовремя диагностировать гипогликемию, что может привести к необратимым последствиям — гипогликемической коме, инсульту, инфаркту и даже летальному исходу [12][13].

Фермент GDH-NAD катализирует окисление первой гидроксильной группы, используя NAD⁺ или NADP⁺ в качестве первичного акцептора электронов. В отличие от GOx и других GDH, окислительно-восстановительный кофактор этого фермента NAD(P) не связан с ферментом, вследствие чего необходимо вводить дополнительный кофактор в электрохимическую реакцию для более полноценной работы фермента [14].

FAD-зависимые GDH получили большое внимание для их потенциального применения в производстве биосенсоров. Эта группа включает оксидоредуктазы, которые катализируют первую гидроксильную группу глюкозы и других молекул сахара, используя FAD в качестве первичного акцептора электронов. Уникальная способность прямого переноса электронов устраняет необходимость в токсичных искусственных электронных медиаторах и делает этот фермент идеальным для биосенсоров глюкозы третьего поколения. Данный фермент не восприимчив к искажающему измеряемые значения влиянию интерферирующих веществ и кислорода, что также является несравненным преимуществом перед его ферментами-предшественниками [15].

Среди наиболее популярных систем мониторинга глюкозы в крови у населения Российской Федерации выделяют такие марки, как Accu-Chek (Roche Diabetes Care GmbH, Германия), Contour Next (Ascensia Diabetes Care Holdings AG, Швейцария) и OneTouch (LifeScan Europe GmbH, Швейцария). Все перечисленные производители указывают в инструкциях к тест-полоскам, что системы соответствуют требованиям стандарта ISO 15197:2013. Также в период с марта по май 2022 г. в Институте исследований и разработок в области диабетических технологий в Германии была проведена оценка точности этих систем на основе ISO 15197-2013, пункт 6.3. Все системы достигли ≥95% результатов в пределах ±15%, как в руках неспециалистов, так и обученного персонала исследования [16].

Относительно недавно в нашей стране появилась отечественная система мониторинга глюкозы Джимейт ЛАЙФ, работающая на основе электрохимического принципа. Она производится в г. Иркутск с середины 2019 г. на заводе полного цикла, с помощью современных технологий, позволяющих производить дешевую, но не уступающую по качеству лучшим зарубежным брендам, продукцию.

При производстве тест-полосок Джимейт ЛАЙФ применяются инновационные технологии карбонового электрода и GDH-FAD. Использование технологии карбонового напыления значительно дешевле использования электродов из драгоценных металлов и, благодаря специфике нанесения, обеспечивает стабильность результатов измерений. Фермент последнего поколения — GDH-FAD не вступает в реакцию с веществами, которые могут попадать в кровь после приема лекарственных препаратов, сахарозаменителей и некоторых других веществ, а также кислородом.

## ЦЕЛЬ ИССЛЕДОВАНИЯ

Целью данной публикации является изучение соответствия точности измерений системы мониторинга глюкозы Джимейт ЛАЙФ требованиям ГОСТ Р ИСО 15197-2015 «Тест-системы для диагностики in vitro. Требования к системам мониторинга глюкозы в крови для самоконтроля при лечении сахарного диабета».

## МАТЕРИАЛЫ И МЕТОДЫ

В процессе проведения экспериментов по изучению основных технических характеристик системы мониторинга уровня глюкозы Джимейт ЛАЙФ использовали 10 глюкометров Джимейт ЛАЙФ (серийные номера HIL1480001-HIL1480010), ланцеты, скарификаторы, тест-полоски GDH-FAD (лоты: Лот 1 (Т3С117), Лот 2 (3Т2С221), Лот 3 (Т2С228)), выполненные по ТУ 26.60.12-001-87060442-2018 регистрационное удостоверение № РЗН 2019/8725 от 23 мая 2022 (производитель ООО «МедТехСервис», г. Иркутск).

Все изделия, согласно инструкции по эксплуатации, входящие в комплект системы мониторинга Джимейт ЛАЙФ сделаны из нетоксичных материалов. Глюкометры Джимейт ЛАЙФ выполняются из АБС-пластика, что делает их ударопрочными, термостойкими и слабо чувствительными к влаге. Экран достаточно крупных размеров и выдает четкие цифры. Устройства работают от литиевой батареи 3 В. Время работы составляет не менее 35 ч или не менее 1000 измерений. Скарификатор имеет пять уровней глубины прокола. Тест-полоски на основе фермента GDH-FAD состоят из трех слоев, пластика, карбоновых электродов и ферментного слоя.

Система измеряет концентрацию глюкозы в крови посредством амперометрического биосенсора глюкозы, встроенного в тест-полоску, путем регистрации серии электрических сигналов, возникающих вследствие реакции глюкозы в пробе крови с метаболизирующей ферментной системой GDH-FAD биосенсора. Уровень электрического сигнала тест-полоски при проведении серии замеров глюкометром преобразуется в значение концентрации глюкозы посредством программного обеспечения устройства и затем отображается на ЖК-дисплее глюкометра. Биосенсорный электрод тест-полоски состоит из карбоновой пасты, основой которой является углерод и органические смолы.

Система Джимейт ЛАЙФ использует технологию «без кодирования». Это означает, что код партии «записан» на каждой тест-полоске и автоматически считывается при введении тест-полоски в прибор. Технология «без кодирования» сокращает риск ошибок в дозе инсулина, который может быть высок вследствие неправильно введенного кода и получением недостоверного результата измерения глюкозы в крови, экономит время для обучения пациентов [17].

Оценку точности системы проводили в период с 05.03.2025 по 06.03.2025 г. в эндокринологическом отделении ОГАУЗ «ИГКБ № 10» г. Иркутска.

Все эксперименты проводили при температуре 23±5 °C.

Тестирование проводили на капиллярной крови.

В качестве референтной методики использовали биохимический анализатор YSI 2900D, производства YSI Incorporated, США. Свидетельство о поверке № С-В/12-09-2024/369911996 от 12.09.2024. Данный прибор был выбран в качестве эталонного, исходя из его технических характеристик (погрешность измерения 2%). Применение YSI 2900D для оценки эффективности использования системы мониторинга глюкозы Джимейт ЛАЙФ выполнено в соответствии с приказом Минздрава России от 09.01.2014 № 2н Приложение 5. п.53.7.

Референтная методика YSI 2900D обладает высокоточными функциональными характеристиками и может быть использована для измерения уровня глюкозы в капиллярной крови вне организма и для оценки медицинского изделия «Система контроля уровня глюкозы в крови Джимейт ЛАЙФ по ТУ 26.60.12-001-87060442-2018 регистрационное удостоверение №РЗН 2019/8725 от 23 мая 2022», производства ООО «МедТехСервис», Россия.

Для дизайна экспериментов по изучению точности измерения системы мониторинга уровня глюкозы Джимейт ЛАЙФ опирались на требования ГОСТ Р ИСО 15197-2015.

Оценка точности системы была проведена в актуальных условиях применения с использованием образцов капиллярной крови амбулаторных пациентов с сахарным диабетом 1 и 2 типа.

## Этическая экспертиза

Согласно постановлению правительства РФ от 30.11.2024 №1684 п. 31 в) для медицинских изделий для диагностики in vitro, медицинских изделий, прошедших государственную регистрацию в соответствии с особенностями, установленными правительством Российской Федерации, и программного обеспечения, являющегося медицинским изделием, получение разрешения регистрирующего органа на проведение клинических испытаний, в том числе с участием человека, не требуется.

## Статистическая обработка

Для проведения оценки точности системы протестировали 600 образцов капиллярной крови, подобранных по соотношению образцов (табл. 1).

**Table:** Таблица 1. Концентрация глюкозы и соотношение образцов для оценки точности системы

Бин №	Соотношение образцов, %	Концентрация глюкозы, ммоль/л
1	5	≤2,77
2	15	>2,77–4,44
3	20	>4,44–6,66
4	30	>6,66–11,10
5	15	>11,10–16,65
6	10	>16,65–22,20
7	5	>22,20

В тех случаях, когда бин не содержал достаточного числа проб свежей капиллярной крови с очень низкими или очень высокими концентрациями глюкозы, они были замещены модифицированными пробами капиллярной крови, в которых концентрации глюкозы были повышены или понижены, чтобы достичь требуемого распределения. Согласно стандарту, критериями минимальных функциональных характеристик точности системы контроля глюкозы являются:

-критерий a: 95% результатов всех измерений глюкозы, выполненных глюкометром, не должны отклоняться от референсных значений более чем на ±15% при концентрации глюкозы ≥5,55 ммоль/л и не более чем на ±0,83 ммоль/л при значениях <5,55 ммоль/л;

-критерий b: не менее 99% результатов измерения должны попадать в зону A и B Согласованной сетки ошибок Паркса для СД1 (рис. 1).

**Figure:**
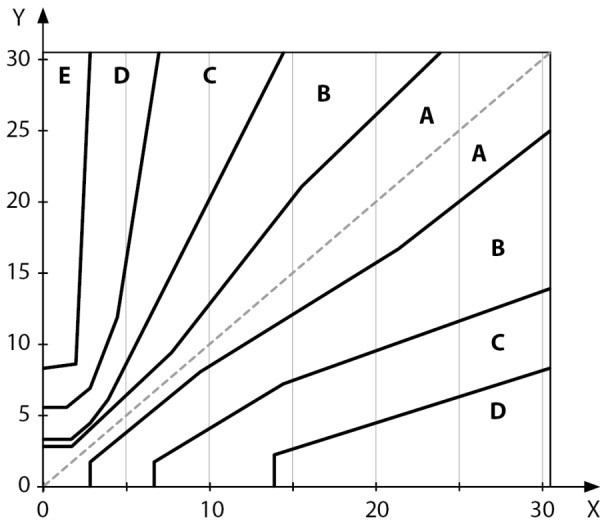
Рисунок 1. Согласованная сетка ошибок, ммоль/л.

Согласованная сетка ошибок Паркса представляет собой результаты опроса 100 эндокринологов, участвовавших в ежегодном совещании Американской диабетической ассоциации (ADA) в 1994 г. Консенсус указанных врачей привел к созданию координат Согласованной номограммы Паркса (рис. 1).

Согласованная сетка ошибок разделена на пять зон, которые определены путем оценки степени риска для пациента. Классификация уровней риска, определенных зонами Согласованной сетки ошибок, представлена в таблице 2 [18].

**Table:** Таблица 2. Определение зон сетки соответствующих ошибок

Уровень риска (зона Согласованной сетки ошибок)	Риск для больного диабетом
A	Не влияет на клинические меры.
B	Изменение клинических мер — незначительное влияние или его отсутствие на клинический результат.
C	Изменение клинических мер — существует вероятность оказания влияния на клинические результаты.
D	Изменение клинических мер — может вызвать серьезные медицинские риски.
E	Изменение клинических мер — может привести к опасным результатам.

Точность системы определяли путем сравнения результатов, полученных с помощью глюкометров Джимейт ЛАЙФ и лабораторного (эталонного) прибора YSI 2900D.

Статистическая обработка результатов выполнена с помощью пакета программ автоматизированной статистической обработки данных Microsoft Excel.

## РЕЗУЛЬТАТЫ

Точность системы. Оценка точности системы показала, что из 216 образцов крови с концентрацией глюкозы ниже 5,55 ммоль/л:

- 88 измерений (40,7%), произведенных с помощью Джимейт ЛАЙФ по сравнению с YSI 2900, находилось в пределах ±0,28 ммоль/л;

- 162 измерения (75%) находилось в пределах ±0,56 ммоль/л;

- 203 измерения (94%) находилось в пределах ±0,83 ммоль/л (табл. 3).

Из 384 образцов крови с концентрацией глюкозы выше 5,55 ммоль/л:

- 244 измерения (63,5 %), произведенные с помощью Джимейт ЛАЙФ по сравнению с YSI 2900, находились в пределах ±5%;

- 352 измерения (91,7%) находились в пределах ±10%;

- 380 измерений (99 %) находилось в пределах ±15% (табл. 3).

**Table:** Таблица 3. Результаты точности системы контроля уровня глюкозы в крови Джимейт ЛАЙФ

Результаты точности системы на концентрации глюкозы ниже 5,55 ммоль/л
В пределах ± 0,28 ммоль/л	В пределах ± 0,56 ммоль/л	В пределах ± 0,83 ммоль/л
88/216 (40,7%) (в пределах ±0,28 ммоль/л)	162/216 (75%) (в пределах ±0,56 ммоль/л)	203/216 (94%) (в пределах ±0,83 ммоль/л)
Результаты точности системы на концентрации глюкозы выше 5,55 ммоль/л
В пределах ±5%	В пределах ±10%	В пределах ±15%
244/384 (63,5%)	352/384 (91,7%)	380/384 (99,0%)
Обобщенные результаты точности системы
В пределах ±0,83 ммоль/л или ±15%
583/600 (97,2%)

Таким образом, из 600 измерений 583 (97,2 %) удовлетворяли критерию a минимальных функциональных характеристик точности системы п. 6.3.3 ГОСТ Р ИСО 15197-2015: (рис. 2, 3):

**Figure:**
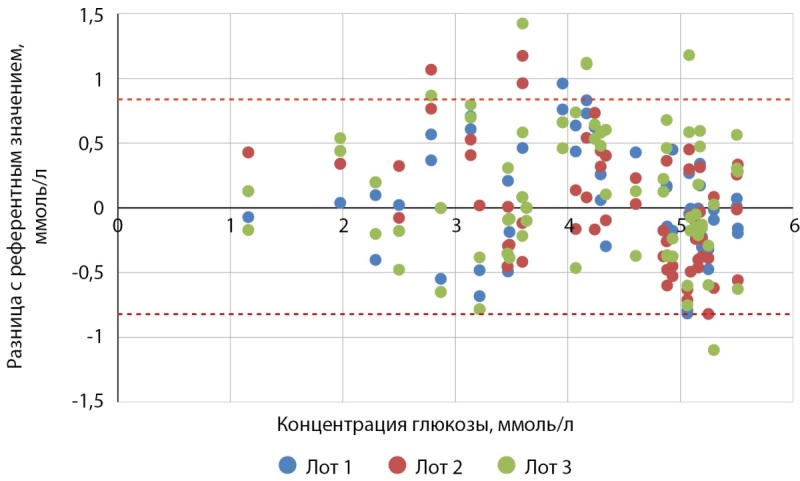
Рисунок 2. Точность системы Джимейт ЛАЙФ для образцов крови с концентрацией глюкозы ниже 5,55 ммоль/л.

**Figure:**
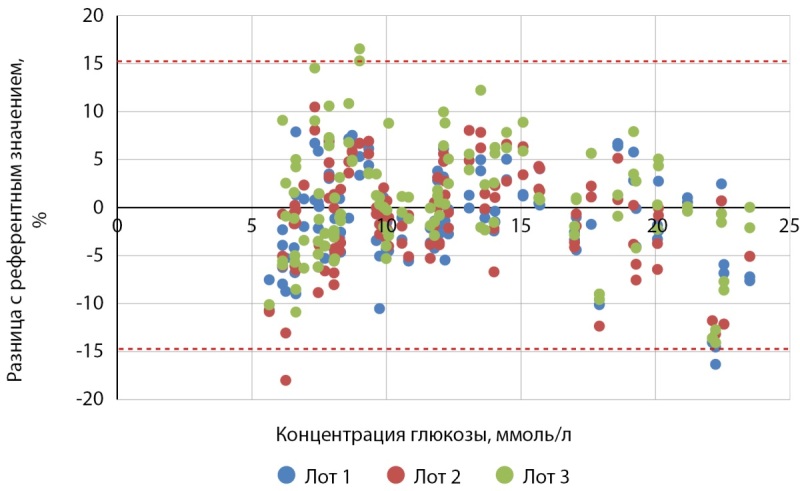
Рисунок 3. Точность системы Джимейт ЛАЙФ для образцов крови с концентрацией глюкозы выше 5,55 ммоль/л.

Все 600 измеренных значений (100%) вошли в зоны A и B Согласованной сетки ошибок (критерий b) (рис. 4).

**Figure:**
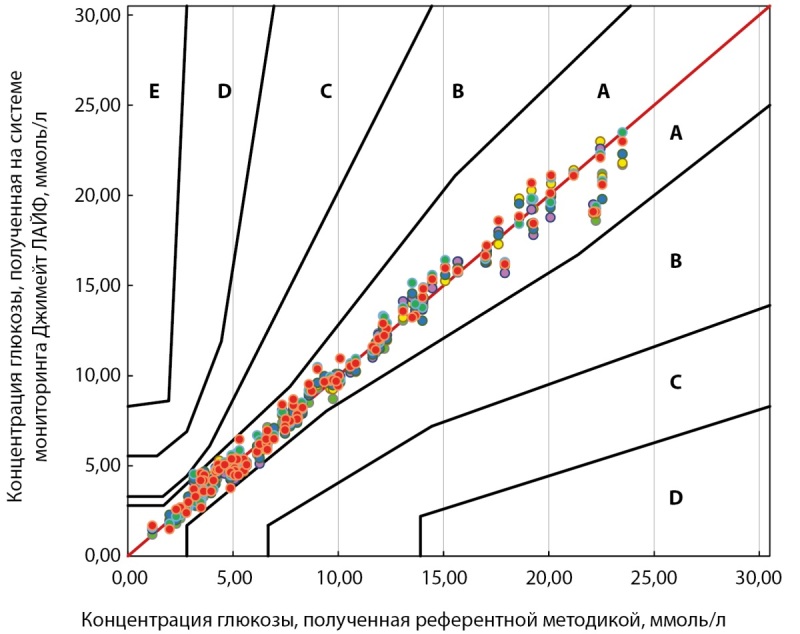
Рисунок 4. Согласованная сетка ошибок измерений, произведенных системой мониторинга глюкозы в крови Джимейт ЛАЙФ, по сравнению с референтной методикой.

Таким образом, в результате проведенных исследований выявили соответствие системы мониторинга глюкозы крови Джимейт ЛАЙФ минимальным функциональным требованиям, предъявляемым ГОСТ Р ИСО 15197-2015 в разделе 6.3 «Точность системы»: точность системы составила 97,2% при минимальном требовании 95%.

## ОБСУЖДЕНИЕ

## Репрезентативность выборок

Репрезентативность выборок, валидность выводов исследования соответствовали требованиям ГОСТ Р ИСО 15197-2015 «Тест-системы для диагностики in vitro. Требования к системам мониторинга глюкозы в крови для самоконтроля при лечении сахарного диабета».

## Сопоставление с другими публикациями

На момент подготовки статьи к публикации другие независимые исследования точности системы мониторинга Джимейт ЛАЙФ найдены не были.

Подобное исследование проводилось для системы мониторинга глюкозы Сателлит, также широко используемой на территории РФ. Оно освещено в статье 2023 г. Дианова с соавт. «Точность измерения и интерференция при самоконтроле гликемии с помощью глюкометра САТЕЛЛИТ® ONLINE у больных сахарным диабетом. Эффективная фармакотерапия»: пределы допустимой системной погрешности измерений глюкозы в крови соответствовали требованиям пункта 6.3 «Точность системы» ГОСТ Р ИСО 15197-2015 — 97,2% отклонений показаний от референтных значений соответствуют зонам клинически верных и безопасных отклонений. Также в публикации этого исследования приводились данные исследований по оценке интерференции влияющих величин и веществ, что также планируется в будущем изучить для системы Джимейт ЛАЙФ с более расширенным списком интерферентов [19].

Исследование точности измерений систем мониторинга глюкозы в крови Contour Next (Ascensia), Accu-Chek Instant (Roche), Medisafe Fit Smile (Terumo) и OneTouch Ultra Plus Reflect (LifeScan) освещено в статье 2022 г. Плеуса с соавт. «User Performance Evaluation and System Accuracy Assessment of Four Blood Glucose Monitoring Systems With Color Coding of Measurement Results»: все исследованные системы измерения уровня глюкозы в крови продемонстрировали достаточный уровень точности, с небольшими различиями между ними. Также в этой статье оценивалась точность измерений четырех систем при использовании их непрофессиональными пользователями, для выявления возможных ошибок при использовании. Непрофессиональные пользователи заполняли анкеты, касающиеся четкости и уместности маркировки производителя, а также удобства использования системы, а также анкету, посвященную цветовой кодировке результатов измерения уровня глюкозы в крови в целом [16]. Такие исследования можно провести в отношении системы Джимейт ЛАЙФ для составления рекомендаций производителю в целях улучшения качества и безопасности при использовании их продукции пациентами.

## Клиническая значимость результатов

Были проведены независимые исследования точности системы мониторинга глюкозы в крови Джимейт ЛАЙФ в реальных условиях использования. В результате было подтверждено соответствие систем Джимейт ЛАЙФ минимальным требованиям стандарта ГОСТ Р ИСО 15197-2015, предъявляемым к точности измерений. Пациенты могут получить достоверные результаты измерений концентрации глюкозы в рамках погрешности, допустимой стандартом ГОСТ Р ИСО 15197-2015, в своей крови при использовании системы Джимейт ЛАЙФ.

## Ограничения исследования

Факторы, которые могли привести к систематическим и случайным смещениям результатов, связанные с проведением этого исследования системы Джимейт ЛАЙФ, сведены к минимуму. Исследование точности системы Джимейт ЛАЙФ, начиная с методов и завершая интерпретацией результатов, проводили согласно требованиям ГОСТ Р ИСО 15197-2015.

## Направления дальнейших исследований

В продолжении работы планируется изучение влияния объемной доли эритроцитов и интерферирующих веществ на точность системы мониторинга глюкозы Джимейт ЛАЙФ.

## ЗАКЛЮЧЕНИЕ

В современном мире технологии не стоят на месте, в том числе и в сфере разработки медицинских изделий. Системы мониторинга уровня глюкозы в крови прошли путь от бумажных индикаторных тест-полосок до высокоточных неинвазивных систем.

Неутешительная статистика по заболеваемости сахарным диабетом, а также технологические и экономические препятствия, влияющие на переход на не- и малоинвазивные измерители глюкозы и приборы непрерывного мониторинга глюкозы говорят о том, что приобретение обычных глюкометров и тест-полосок будет еще долгое время актуальной потребностью для населения. На данный момент глюкометры и тест-полоски занимают первое место по продажам среди диагностических приборов, и объемы продолжают расти.

Важно понимать, что точность измерения концентрации глюкозы в крови зависит от уровня технологии и качества прибора и тест-полосок. Система должна соответствовать минимальным требованиям стандарта ГОСТ Р ИСО 15197-2015. Тестирование системы Джимейт ЛАЙФ показало, что точность системы мониторинга глюкозы Джимейт ЛАЙФ полностью соответствуют стандарту и превосходят минимальные требования.

## ДОПОЛНИТЕЛЬНАЯ ИНФОРМАЦИЯ

Источники финансирования. Работа выполнена по инициативе авторов без привлечения финансирования.

Конфликт интересов. Авторы декларируют отсутствие явных и потенциальных конфликтов интересов, связанных с содержанием настоящей статьи.

Участие авторов. В.А. Быбин — подготовка образцов глюкометров и тест-полосок и проведение тестирования, математическая обработка результатов тестирований; Н.М. Быкова — проведение тестов на капиллярной крови, сбор и обработка материала для публикации, математическая обработка результатов тестирований; Е.А. Харитонова — сбор и обработка теоретической части статьи, математическая обработка результатов тестирований.

Все авторы одобрили финальную версию статьи перед публикацией, выразили согласие нести ответственность за все аспекты работы, подразумевающую надлежащее изучение и решение вопросов, связанных с точностью или добросовестностью любой части работы.
